# Palliative versus corrective surgery in new cardiac programs

**DOI:** 10.1186/1749-8090-8-S1-O308

**Published:** 2013-09-11

**Authors:** K Fenton, S Hernandez, C Duarte, N Berrios, W Novick

**Affiliations:** 1 International Children's Heart Foundation, Memphis, TN, USA; 2 Asociacion Programa Corazon Abierto, Managua, Nicaragua

## Background

In developing countries where pediatric cardiac surgery is in its infancy, palliative operations that can be done without cardiopulmonary bypass are often perceived to be a better surgical option than open heart surgery, which is thought to be complicated and high risk. We sought to evaluate outcomes from the first five years of a congenital heart surgery project in Nicaragua.

## Methods

Retrospective review was conducted of all children undergoing palliative and reparative (open or closed) cardiac surgery between January 2007 and December 2012.

## Results

A total of 301 primary cardiac operations were performed. Overall early mortality rate was 6.0% (18 deaths). There were 12 deaths in 31 palliated children (39%), and 6 deaths in 270 repaired patients (2.2%, p<0.001). Mortality was highest (8/20, 40%) in patients undergoing “Stage 1” type palliation (systemic to pulmonary artery shunt or pulmonary artery band).

## Conclusions

Although the surgery itself is apparently logistically and technically easier, mortality rates are high in palliative operations; in fact, the difference in results between palliative and reparative surgery is higher than that commonly reported for established programs in classification systems such as RACHS. Even early on in program development, the lowest risk option for any given patient may often be complete repair.

